# Experiential training course on spirituality for multidisciplinary palliative care teams in a hospital setting: a feasibility study

**DOI:** 10.1186/s12904-024-01341-6

**Published:** 2024-02-10

**Authors:** Silvia Tanzi, Giovanna Artioli, Elisabetta Bertocchi, Giulietta Luul Balestra, Luca Ghirotto, Mario Cagna, Filippo Laurenti, Simona Sacchi

**Affiliations:** 1 Palliative Care Unit, Azienda USL-IRCCS, Reggio Emilia, Italy; 2 Department of Medicine and Surgery, University of Parma, Parma, Italy; 3 Qualitative Research Unit, Azienda USL-IRCCS, Reggio Emilia, Italy; 4 ASL4 Liguria-Chiavari, Chiavari, Italy; 5 Fondazione Luce Per La Vita, Rivoli, Turin, Italy

**Keywords:** Palliative care, Spiritual care, Oncology, Interactive learning, Complex intervention, Comprehensive analysis

## Abstract

**Background:**

There is widespread agreement about the importance of spiritual training programs (STPs) for healthcare professionals caring for cancer patients, and that reflecting on one’s spirituality is the first step. Health professionals (HPs) working in hospitals must develop this dimension to guarantee the quality of life as well as spiritual and emotional support. In this paper, we propose a possible training format for hospital professionals and assess its implementation.

**Methods:**

This is a phase 0-I study that follows the Medical Research Council (MRC) framework. The program was implemented for hospital palliative care specialists. The program included one theory lesson, three spiritual interactions, four pieces of reflective writing, and two individual follow-up sessions for each participant. The evaluation was performed quantitatively according to the MRC framework and qualitatively according to Moore’s framework with data triangulation from interviews, reflective writings, and indicators.

**Results:**

The program was implemented for palliative care physicians, nurses, psychologists, and bioethicists according to the plan, and the program components were highly appreciated by the participants. The results suggest the feasibility of a training course with some corrections, regarding both the components of the training and organizational issues. The qualitative analysis confirmed a shift in the meaning of the themes we identified. The trainees went from intrapersonal spirituality to interpersonal spirituality (engagement with the other person’s spirituality, acknowledging their unique spiritual and cultural worldviews, beliefs, and practices), with colleagues, patients, and people close to them. The training had an impact on Moore’s Level 3b.

**Conclusions:**

Spiritual training for hospital professionals working in palliative care is feasible. Having time dedicated to spirituality and the ongoing mentorship of spiritual care professionals were suggested as key elements. The next step is increasing awareness of spirituality from our hospital reality and creating a stable competent group (with nurses, chaplains, nuns, counselors, etc.) with the support of the management.

**Supplementary Information:**

The online version contains supplementary material available at 10.1186/s12904-024-01341-6.

## Background

Palliative care encompasses spiritual care, considered as its intrinsic and essential component to such an extent that the definition of palliative care includes spiritual care [1]. Spiritual care plays a central role in the holistic construct of palliative care [2]. Caring for the spiritual dimension leads to better outcomes for patients and their families [3].

The European Association of Palliative Care (EAPC) white paper defines spirituality as a multidimensional concept: “Spirituality is the dynamic dimension of human life that relates to the way persons (individual and community) experience, express and/or seek meaning, purpose and transcendence, and the way they connect to the moment, to self, to others, to nature, to the significant and/or the sacred” [4].

The EAPC white paper addresses the issue of spiritual care education for all palliative care professionals, positing that better preparation can help healthcare providers avoid being impeded by their fears in attending to patients and families. The white paper “encourages and facilitates high quality, multi-disciplinary, academically and financially accessible spiritual care education to all palliative care staff” [4].

Working on personal spirituality is essential for palliative care professionals, and some authors suggest that it is even more important than participating in training on spirituality [5].

The Interprofessional Spiritual Care Education Curriculum (ISPEC^©^) was launched in 2018 in the United States and then developed in 16 other countries [6]. The goal of ISPEC^©^ is to train health professionals and chaplains in all clinical contexts so that interprofessional spiritual care can be fully integrated and the spiritual distress of patients treated by all team members. The model this curriculum refers to was based on a generalist-specialist model derived from a consensus on spiritual care between physicians who provide generalist spiritual care and trained chaplains who provide specialist spiritual care; in the generalist-specialist spiritual care model, all clinicians address spiritual issues and work with trained chaplains who are spiritual care specialists.

A recent systematic review [7] contributed to the investigation of spiritual training programs (STPs) for health professionals (HPs), providing insights into spiritual care training conducted over the last ten years. Features that facilitated training included the involvement of chaplains, opportunities for practice and reflection, online training, and the support of management.

Practical training was always advocated, with a good alternative to using patients in training courses identified as the practice of spiritual interactions between peers [8]. Coaching was found to be an effective method in the education of HPs [9, 10], as well as reflexivity [11] in groups and among peers, in particular for the development of non-technical skills [12], with long-term supervision [13]. A multidisciplinary approach was recognized as indispensable for all spiritual training, as expressed in the title of the study by Jones et al. “Spirituality is everybody’s business” [14]. All HPs should have some tools to increase awareness of the spiritual dimension and work with it as a team. Ethical and legal issues were also recognized as spiritual care competencies in comprehensive spiritual training [15] but were found to be less represented topics [7].

However, few experiences have been described for specialist palliative care services (SPCSs) in hospitals, with the majority of studies involving specialist nurses [16–18]. In van de Geer’s study [19], non-specialist palliative care professionals working in hospitals were involved, and the authors found that spiritual care training in palliative care for HPs in teaching hospitals can have a positive effect on staff attitudes and competencies.

Lastly, to our knowledge, Italy has not yet produced any training programs of this nature.

Beginning from the lack of courses on the spiritual dimension for hospital specialist services in palliative care and the lack of courses on spirituality in our specific cultural context, we developed a novel training program to test its feasibility and acceptability by a group of multi-professional palliative care HPs. The course was implemented with components that the literature suggests should be included, such as experiential learning techniques [8], peer interaction, one-to-one coaching [9] with spiritual specialists [10], and methodologies that stimulate self-reflection [11] with an extended duration supervision [13].

The specific objectives of the study were:

1. Implementation of a training intervention for health professionals (doctors, nurses, psychologists, bioethicists) to increase their knowledge and skills regarding spirituality.
2. The qualitative evaluation of the training course by applying Moore’s expanded outcomes framework [20].

The training was developed, implemented, assessed, and evaluated as a complex intervention, according to phase 0-I of the Medical Research Council (MRC) framework [21].

## Population and methods

### Population and context

The study was carried out at the Arcispedale Santa Maria Nuova Hospital in Reggio Emilia from November 2021 to November 2022. This is a 900-bed Italian research hospital, certified as a Comprehensive Cancer Centre by the Organization of European Cancer Institutes (OECI). The Palliative Care Unit (PCU) is a specialist hospital-based unit with no beds whose mission is to perform clinical, training, and research activities in palliative care. The PCU provides specialist consultations in the hospital as well as in an outpatient clinic for oncological patients and their relatives.

The unit was established in 2013 and at present includes three senior physicians and three advanced practice nurses. Five psychologists from the hospital’s Psycho-Oncology Unit work with the PCU through clinical consultations and with responsible for PCU staff training, as well as carrying out research and training in palliative care. The Bioethics Unit (BU) comprises two bioethicists. The purpose of the BU is to assess and promote the quality of care for patients, family caregivers, and healthcare professionals through research projects, educational programs, and ethics consultation activities.

All the professionals of the PCU, Psycho-Oncology Unit, and BU were enrolled in the study. They were contacted by e-mail and none of them refused to participate.

The training was conducted by two spiritual care professionals (SCPs). F.L. works mainly in hospices and is a member of the Scientific Committee of the Italian Journal of Palliative Care (*Rivista Italiana di Cure Palliative*). M.C. works both in hospices and in a general hospital with acute and intensive care units and is co-author of the Core Curriculum for Spiritual Care published by the Italian Society for Palliative Care (*Società Italiana di Cure Palliative*) [22].

### Methods

This was a Phase 0-I study. According to the Medical Research Council (MRC) framework for complex intervention [21], a training program was developed considering the limitations of the existing literature on spiritual care training for hospital SPCSs in Italy.

The spiritual assistants were always online due to COVID restrictions and the HPs always participated in person.

The program layout.

1. One theory lecture (HPs present in the classroom, spiritual assistants online), an interactive meeting with the SCPs to inform the participants about the program content (4 h). The goal of this lesson was to introduce the HPs to the concept of spirituality (being mindful of the clinical setting). The approach was mixed: partly with lecture format and partly with group work.
2. Meetings between SCPs and HPs in pairs. Each pair of HPs participated in three meetings and then two individual follow-up meetings 3 and 6 months after the beginning of the training course. Before starting the meeting (warm-up activity), each pair of HPs shared: ‘what spirituality means to me’. Each meeting had a specific purpose:
  a During the first meeting (HPs present in the classroom, spiritual assistants online), the aim was for participants to define what spirituality means to them and what their spirituality consists of (45 min). Some guiding questions included: Do you feel spiritual? What is the importance of spirituality in your life? What importance does it have to you in terms of community? The questions were inspired by Christina Puchalski’s FICA tool [23].
  b During the second meeting (HPs present in the classroom, spiritual assistants online), the aim was for participants to reflect on what can contribute to developing their spirituality (30 min). Some questions included: What form did your spirituality take as a child/teenager? Is there anything from that time that has not occupied time and space in your life since then? If you think about the significant people in your life, what comes to mind concerning the theme of spirituality?
  c During the third meeting (HPs present in the classroom, spiritual assistants online), the aim was for participants to define which measures can be implemented to maintain their spirituality and help it grow (30 min). Questions from the SCPs included: How do you intend to take care of your spirituality from now on? What is empty and what is full in your spiritual life?
  d Two online follow-up meetings (HPs present in the classroom, spiritual assistants online) involved guided meditation and a free space for discussing the previous meetings. In particular, the first follow-up focused on the participants’ spirituality, and the second one on interpersonal spirituality (with patients and colleagues).

3) Individual reflective writing and discussion in pairs (both professionals present in the classroom) After participating in the meetings with the SCPs, each participant wrote a reflective journal on the experience, answering a series of pre-defined questions in writing (15–20') based on the literature [24]. After writing about their experience, the two HPs met and shared any details they considered relevant, reflecting further on their experience.

**Fig. 1:**
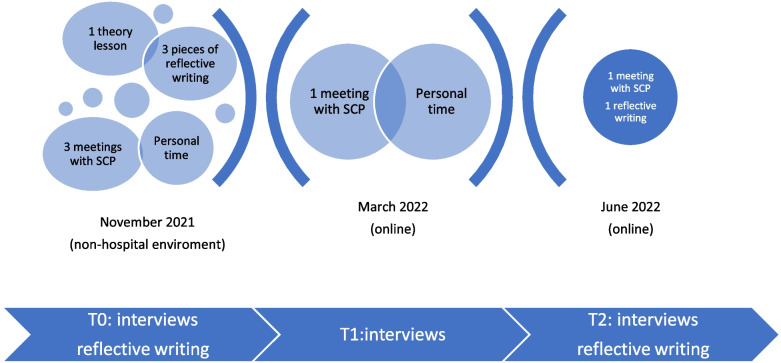
The training program

The training program is illustrated in Fig. 1.

The educational model used by the SCPs was based on guidelines and recognized core curricula ([6, 22]; https://www.aamc.org/media/24236/download, https://advancingexpertcare.org/position-statements/spiritualcare) together with personal self-reflection and spiritual growth.

All the training was carried out in an environment outside the usual place of work. For the duration of the various activities, the participants were invited to maintain an atmosphere of silence and reflection, and background music was played to promote relaxation and personal concentration. The music room was used for trainees who were waiting to take part in the meetings and/or reflective writing and for those who had already completed them. The meetings with SCPs were carried out via a virtual platform, in which the SCP instructors were online and the training participants met in person.

We considered the training as a complex intervention with assessment [21]. The overall assessment was performed using a mixed-method evaluation with concurrent triangulation. The concurrent triangulation consisted of a qualitative and quantitative collection of data in the same period, in one subsequent separate analysis and lastly in the integration of the results during interpretation [25].

We used data triangulation to increase the validity of the study results.

We decided to conduct a before-during-after evaluation of the training, inspired by Moore et al.’s expanded outcomes framework [20]. Moore’s framework consisted of 7 progressive levels of learning by practitioners. We focused on Moore’s 3B level because it seemed congruent to evaluate the impact on personal performance.

- Participation—Level 1 – The number of HPs who participated in the training.
- Satisfaction—Level 2 – The degree to which the participants’ expectations were met.
- Learning—Level 3A – Declarative Knowledge – (Know) The degree of knowledge that participants declared they have learned.
- Level 3B – Procedural Knowledge – (Know-how) The degree to which the participants show understanding of *knowing how to do.*

Information on the objectives achieved or not achieved was collected for each component of the program, and semi-structured interviews at T1 were conducted to collect feedback on program components (see Table 1 training component feedback). We considered the program feasible if:

- the components of the training course were appropriately identified as active components of the intervention
- the program was completed as planned for the three hospital units and all the participants.

**Table 1:** Feasibility table; evaluation of training components

Components	Implemented	Duration as planned	Timeline as planned	Trainee attendance	Qualitative feedback from participants (codes) T1	T2
Theory lesson	yes	yes	yes	100%	Very much appreciated; limited content but clear (1,2,8) and “sensitive” (6) Need more references (3)	Useful to reorganize some concepts (code 11, code 8) No improvement in knowledge with this lesson (code 10)
Spiritual interactions between SCPs and 2 HPs	yes	yes	yes	100%	The most cited words were significant (1,2,4,8,12) and very touching (2,6). Some trainees expressed an initial embarrassment (3,4,8) due to the intimate and sensitive nature of the topic The sharing of their personal views about spirituality with their peers without judgment and with attentive listening by the SA was perceived as very natural and rich (5,6,8) The different style of the SA was very much appreciated (11,12)	The discussions in pairs and then individually with a teacher was very moving, and allowed us to work on this dimension (code 1) the discussions with others were more effective than self-reflection (code 4,8) exchanges with colleagues were appreciated, which was not a given (code 4, 7)
Spiritual interaction between SCPs and individual HP	yes	yes	yes	100%		felt that the follow-ups were very useful, because they didn’t require any initiation by the trainee (code 2)
Reflective writing	yes	yes	yes	100%	The majority of trainees criticized this modality saying it is too rigid and not spontaneous (1,3,4,5,6,7,9). Others found it useful to discover some insights and share them with colleagues (8,11,12)	felt reflective writing was too rigid and not spontaneous (code 11,single comment)
Discussion between peers on RW	yes	yes	yes	100%	The trainees who talked about this aspect all gave positive feedback about the discussion, the perceived listening by colleagues, the help in find the meaning of the RW (2,3,4,5,8,11,12) Only one (10) didn’t find it so useful	X
Personal time	yes	yes	yes	100%	All trainees appreciated the personal time and used it according to their own feelings: listening to relaxing music (1), praying (4), eating slowly (3), meditating (5), walking in nature (6), reading spiritual lectures (7), taking photos (8).Only 2 trainees criticized the silent time because without any framework they found it difficult to maintain silence, and it didn’t not work for them (8,10)	moments of silence or exercises (too standardized) were less appreciated (code 4, 11) felt that the moments of emptiness were an opportunity for enrichment when you are more open to understanding everything (Code 6,8,12)
External context	yes	yes	yes	100%	Highly appreciated by 8 trainees, for the natural environment and for the day-long duration. One trainee also underlined the respect for other people to maintain a quite space (11)	x
Duration		yes	yes	100%	8 trainees talked about the duration, which they were happy with. Two, who had the spiritual meeting at the end of the day, found that they had too much free time to fill (10,6)	x

### Data collection and analysis

We used longitudinal qualitative interviews to assess participant satisfaction and gained knowledge to which we added the reflective journals that the participants wrote as part of the training program.

Thirty-six interviews were conducted, and 35 journals were produced and analyzed.

The researchers were one male (LG) and the others female.

The median duration of the interviews was 58 min (range 21–92 min).

The convenience sample of all the HPs belonging to the three units was selected, and no one refused to participate.

All the participants were interviewed before the training started (see Additional file 1, interview T0) to gather information on their perceived training needs in the area of spirituality and develop the program accordingly (see Table 2). Then, we planned further interviews three months (see Additional file 2, interview T1) and six months (see Additional file 3 interview T2) after the beginning of the training.

**Table 2:** Trainers ‘expectations al T0

Themes (codes)	Quotations (codes)
To better understand one’s own spirituality (2,3,5,7,8,10,11)	…to understand if my idea of spirituality, what I told you, can be considered spirituality or simply a way of life (2) to help me understand my personal “stuff” better (3)
Knowing how to evaluate / recognize / alleviate the spiritual suffering of patients (2,7,4,8,9,10,11)	“Spirituality can have a significant effect on the patient. How can I use my idea of spirituality as a resource and apply it to patient suffering?” (2) “I expect enrichment from a professional point of view” (7)
Being able to discuss a topic like this with other people (3,7)	“I am very interested in the experience of dialogue…as I said before… it is something that I lack experience in and I am not confident with it. I really prefer to listen” (7)
No expectations (1,6)	whatever happens will be useful (1)
Training intended as enrichment regardless of the application (6,12)	I don’t think my expectations are so important, but what I can learn is still important (12)

Codes refer to interviews number

We pre-planned a simple interview guide for T0 (see Additional file 1).

The interview guide was not piloted. All the interviews were audio recorded.

To ensure internal consistency and to be able to evaluate changes in certain areas, the identified topics formed the basis for the subsequent interviews. Furthermore, the interviews at T1 and T2 also involved some questions regarding the training, suggestions on how to adjust the program (see Additional file 2), the participant’s perspective on the training program, and additional suggestions for any future redesign of the program (see Additional file 3).

Additional file 4 shows the participants with their codes.

Two female researchers conducted the interviews: GA, an expert research nurse, and GLB, an anthropologist and qualitative research expert, both active in-hospital researchers.

To assess participation, we gathered quantitative data from the attendance records. We considered attendance of at least 75% of the training hours for each participant as a cut-off. We then calculated the attendance percentage.

The T0 interviews were recorded and transcribed verbatim, and the participants’ training expectations were identified. The other interviews were also recorded and transcribed verbatim.

Finally, we organized a meeting as requested by participants to get feedback from the SCPs. In that meeting, we checked the results with the participants.

The final dataset consisted of interviews, journals, and trainees’ validation of the results.

The data were collected in the workplace, and no one was present besides the participants and the researcher. No field notes were made during the interviews.

We analyzed the interviews from each time point using an inductive approach, employing the framework method (FM) ([24]; https://www.aamc.org/media/24236/download). This analytic approach was appropriate for allowing themes to emerge and enabling inter-coder agreement. By immersing themselves in the data (familiarization), the researchers developed a deep understanding of the content. Then they created a set of initial codes by systematically labeling data segments relevant to the research question. These codes were grouped into categories or themes, creating a working framework. This framework is usually a matrix with rows representing cases or participants and columns representing themes or codes.

ST, EB, and SS independently analyzed the transcripts by repeatedly reading the texts, gradually labeling the segments of a subset of the interviews with codes, and grouping them into themes and macro-themes. The researchers discussed any disagreement, and a final data allocation was determined. LG provided supervision throughout the whole process. Data saturations were discussed.

This shaped the FM, which was applied to the remaining interviews. The application of the FM to the interviews considered how the meanings between T0, T1, and T2 changed and differed within the macro-themes. So, the FM gave researchers a longitudinal perspective, highlighting recurrent and evolving themes in interviews at different times and identifying any differences in meanings and/or perspectives. No software was used to manage the data.

Furthermore, GLB analyzed the reflective journals for each participant using the FM. This process allowed triangulation of the data and consolidation of the results.

### Rigor and reflexivity

Regarding reflexivity, GA had no prior contact with participants. Two palliative care physicians [ST, SS] were both trainees and analyzed the interviews as palliative care nurses [EB]. They had contact with trainees because they work daily with the bioethics and psycho-oncology units. They had previous experience in conducting and analyzing training programs in palliative care because providing training courses for hospital staff is part of the mission of the palliative care unit.

We believe a lot in the importance of the spiritual dimension and our involvement in the course was very high, we were very motivated.

The fact that three of us (ST, SS, EB) analyzed the results may influence positive interpretations.

Three external researchers (LG, AG, GLB) ensured the rigor (as expressed in the method, see Methods section).

The good relations with the colleagues participating in the training certainly helped.

GLB, who has a background in anthropology, together with LG, who is an expert in qualitative research, served as external experts during the whole research process. LG was the chief of the Qualitative Research Unit. Both had no prior contact with the trainees.

## Results

### Developing the spiritual training program

From the analysis of the existing literature and the interviews with the participants at T0 (see Tables 1 and 2), the course was implemented both with trainees’ suggestions and with components that the literature suggests should be included: experiential learning techniques [8], peer interaction, one-to-one coaching [9] with spiritual specialists [10], and methodologies that stimulate self-reflection [11] with an extended duration supervision [13].

### Quality assessment of the program and indicators

The procedure for assessing the quality of the program included a list of indicators covering all of its components (see Table 1). Concerning the theory lesson, spiritual interactions, RWs, having personal time, staying in an external context, and discussion between peers, a 75% minimum attendance rate was estimated by researchers to be reasonable, which is consistent with the study aims.

### Preliminary assessment of the program: the evaluation system

#### Quantitative feasibility data

The training program was implemented as planned. Twelve participants attended the training, representing the 3 units of PCU, Psycho-Oncology, and BU at our hospital. There were 4 psychologists, 3 nurses, 3 physicians, and 2 bioethicists.

Attendance was 100% of the training hours for all participants.

##### Global feedback on the training

Qualitatively, most of the trainees were happy with the duration of the training, though two of them who attended meetings at the end of the workday suggested some changes.

One comment was that the theory lesson was not detailed enough, and there were several suggestions to add some concepts and a reading list between T1 and T2 and for the future.

Most of the trainees criticized the reflective writing, saying that it was too rigid and not spontaneous (see Table 1 for details).

The T1 and T2 interviews gave rise to some suggestions on how to modify the ongoing training and any future training. Some references and a reading list were provided by the SCPs and a final face-to-face meeting was planned in November 2022, six months after the last online follow-up.

The trainees’ expectations were fulfilled, according to the final meeting to validate the results.

##### Organizational issues

Two trainees out of twelve criticized the silent time/personal time because, without any framework, they found it difficult to maintain the silence.

The follow-up sessions were very much appreciated.

Several trainees requested to continue and intensify the monthly exchanges with SCPs in the future (see Table 3: suggestions for improving the training course from interviews at T1 and T2).

**Table 3:** Suggestions to improve the training course from interviews at T1 and T2

T1	Improved during the course	T2
Getting feedback from SCPs at the end of the day	No	confirmed
Planning a monthly appointment like this one	No	confirmed
Having some references to study/check	Yes	confirmed
Changing the order of spiritual interactions during the day	No	Having experiential training with patients
Having a warm-up exercise always before the interactions	No	The constant presence of SCP in daily work
Having more time dedicated to theoretical knowledge	No	Avoiding online meetings

#### Qualitative data

Analysis of the semi-structured interviews before the training, after the 3 days of training, and at the end of the follow-up sessions, along with analysis of the reflective journals, led us to identify four overarching themes (Table 4): (1) What is spirituality? (2) Getting spiritual experience at work, (3) Spirituality and the need for nourishment, and (4) Self-reflection on one own’s spirituality.

**Table 4:** Meaning shift from T0 to T2

Sub-themes T0	Themes T0	Themes T1	Sub-themes T1	Themes T2	Sub-themes T2
**What is Spirituality?**					
S. is the cultivation of personal values S. is something intimate / personal / private S. as self-knowledge	Personal concept of spirituality	Listening to oneself	Spiritual dimension was confirmed	Recognition of a personal background	Personal component of s The starting point Reconnection with one’s own spirituality Spaces for oneself to cultivate s s. confirmed spiritual practices
The substance of S The spiritual experience is in life itself S. as a dynamic dimension	S. as Life	Listening to others	Spirituality is listening to oneself and to others	Others	Seeing others as they are
Five Senses are S Connection with nature	Nature	S. as meaning	*S. is sense, meaning*	S. is pervasive	Pervasive concept S. as a search for meaning transcendent
**Getting spiritual experience at work**					
S. is the connection with others, feeling oneself in the relationship with others	Experiencing s. as a connection and exchanging with each other	Experiencing s. as greater attention to the s. of the patient	Being able to identify the patient’s spiritual needs	Spiritual care tools	
Tolerance, not judgment, closeness to others	Openness without judgment, including in the role of carer	Listening to oneself helps listening to suffering and death	Paying more attention to the s. of patients thanks to their awareness of their own s Consistency between professional values and approach to s	Professional area as a space to cultivate spirituality	S. in the profession Connection
Healing as a tool to nourish one’s own spirituality S. is felt at work Taking care of yourself at work / taking care of your spirituality at work	The workplace is the place to cultivate s	Team connection to the s. of others (colleagues, patients)	Connection to each other (friends / colleagues / patients) Closer contact with colleagues during the course Team listening to the patient’s spirituality	Circularity of s. (colleagues, patients, close people)	Desire to investigate spiritual dimension even in people close to you S. it must be shared / discussed with colleagues
**Spirituality and the need for nourishment**					
Spiritual practices Training your spirituality	Taking care of your spirituality	More attention to oneself. Active need for nourishment	New forms of nourishment for that person Feeling the need to nourish their own spirituality		
	Well-being perceived when approaching the spiritual dimension	Renewed attention to one’s personal spirituality	Having acquired greater awareness of the spiritual dimension Spiritual practices	Need to find one’s own way of working on oneself	
	fatigue/sense of fatigue of operators	Struggle to find time and space to cultivate spirituality		Theoretical purpose to nourish, not applied	
**Self-reflection on one’s own spirituality**					
	Understanding the use of your spirituality	Acquisition of greater confidence with one’s s	Idea of s. confirmed Widening and greater confidence with respect to the vision of s	gratitude	Expansion
		Questioning of one’s spirituality		Keeping spiritual awareness high	Familiarity with the topic Spiritual awareness
				Difficult to apply in workplace	fatigue/sense of fatigue of operators

*S.* Spirituality

These themes emerged with different meanings and nuances (defined within the sub-themes) about pre-training and post-training data collection.

##### ***Theme 1: What is spirituality?***

As the first result of the training, we observed a shift in meaning from what we called “the personal dimension” of spirituality, made up of specific knowledge and personal values, to a spirituality consisting of “listening to oneself” (T1) and finally to the acknowledgment of the “personal background” that influences a person’s approach to spirituality (T2).

This emerging theme was consistent with the adopted FM theme “understanding the concept of spirituality” (in brackets interview number, interview label number).

“I recognized my colleagues’ and my own spiritual aspects more thanks to the course” (code 2.2 T1)

“Spirituality is an awareness of oneself” (code 8.5 T1)

From T0 to T2, the personal component of spirituality acquired more recognition, and we observed a development in awareness of the HPs’ spiritual dimension, which was “reawakened” at the end of the course.

“The course ‘awakened’ feelings I had experienced in the past” 12.8 T2

“I discovered religion again through meditation” 11.12 T2

“The course reopened the door to the spirituality I experienced 20 years ago” 7.1 T2

Spirituality, perceived as very tangible and as “Life Itself” at T0, acquired an attitude of openness to other people (“Listening to others” T1) and even more at T2, to the absence of judgment in relationships with peers (“Others as they are”).

The FM theme “gaining awareness of the importance of understanding one’s spirituality before addressing the spiritual needs of others” was confirmed by these results.

“Spirituality is a dialogue with others, not just with yourself” 7.4 T1

“It’s important to listen to yourself to be able to listen to others” 8.4 T1

“The face-to-face meetings improved our friendships; in an environment outside the hospital, they have allowed us to ‘see’ each other as we are” 12.6 T2

Moreover, even though the perception of spirituality was quite broad (including nature and experienced through the five senses) from T0, at T2 the concept of spirituality had become “pervasive”, often including a search for meaning and connection with God and faith.

“I understood that everything is spirituality, it belongs to the individual, it is personal and not the same for everyone” (code 5.5 T2)

*“…the seasons, the sun, the fresh air when I go for a run… it’s laughing, crying, hugging my children, cooking for them. Spirituality is everyday life, being present in actions with body and soul” (code 6.4 T2)*

##### ***Theme 2: Getting spiritual experience at work***

This training course involved units specializing in palliative care, where the experience of spirituality is often already felt in relationships at work. In this way, it seemed that what was already there (“experiencing spirituality as a connection with others”) was strengthened after the course in what we called a “greater focus on patient spirituality”. (T1)

“I understood how an awareness of your spiritual dimension leads to identifying it more in others” 3.18 T1

“After the course, I focused more on listening to patients”, 1.6 T1.

Consequently, at T2 the professionals started to express the need for more “spiritual care tools”, some of which (e.g., making time, and active listening) they found and experienced personally during the training.

*“The IPOS* [Integrated Palliative Care Outcome Scale] *(PROMS* [patient-reported outcome measures] *could be used as a trigger tool to talk about spirituality (search for the meaning of life)” (code 12.14 T2)*

“…What is important to the patient? What makes the patient feel good? These are trigger questions for the spiritual dimension” (code 9.30 T2)

“The course has provided me with useful resources that I will draw on” (code 8.1 T2)

At T0, participants identified the workplace as a place to cultivate spirituality and take care of themselves. After the 3-day course, they recognized a team connection to the spirituality of colleagues and patients.

“After the course, we worked with the team on patient spirituality, everyone doing their bit” 1.7 T1

“I understood how the spiritual dimension arises from mutual exchange” 12.3 T1

At T2, this openness to the spirituality of their peers emerged and, at the same time, ongoing dialogue and sharing with colleagues and those closest to them were identified, a phenomenon we called the “circularity of spirituality”.

“The “circularity of spirituality” … I understood the importance of these inside-out and outside-in aspects of spirituality” (code 8.13 T2)

“…*deepening your spirituality is like a bridge that connects us to patients as human beings and care providers*” (code 6.36 T2).

Finally, looking at the spiritual experience at work, we observed a shift from a position of “openness without judgment, including in the role of care providers” to a deeper listening to patients about their suffering (at T1). Gradually, throughout the process and fully at T2, the trainees perceived themselves as spiritual tools through deep listening and connection.

“Being in touch with your spirituality also allows you to be in touch with suffering and death” (code 8.3 T1)

*“For those that are suffering greatly, reconnecting with their human nature, including through the relationship with the health care professional is precious, divine” (code 9.33 T2).*

“Connection” and relating to others is a way to be a “spiritual tool” in the professional context of palliative and cancer care.

##### ***Theme 3: Spirituality and the need for nourishment***

Many of the trainees already cultivated some spiritual practices, nurturing their spirituality.

After the course, the increased attention to their spirituality triggered the need for nourishment, including through experiencing new forms of spirituality and new practices.

Examining one’s spiritual dimension was recognized as giving a feeling of well-being and the course renewed the participants’ attention to their spirituality.

“I felt the need to talk about and nurture my spirituality by sharing it with others” (code 1.9 T1)

“In the practice of silent retreats, in observing, in taking some time and space without having to fill in every gap” (6.13 T1)

“I’ve re-discovered an idea of spirituality linked to recollection, prayer, contemplation, listening” (8.7 T1)

This increased self-awareness of participants’ spiritual dimension led to what we termed the “Need to find one’s way of working on oneself” at the end of the course.

“Everyone has to work on himself, in a personal way, in his way” (code 9.17 T2)

“By now I possess this spirituality and it’s up to me to decide whether to develop it or not” (code 8.22 T2)

##### ***Themes 4: Self-reflection on one’s spirituality***

The participants mainly started the course with a utilitarian view of spirituality, seeking to “understand the use of spirituality”.

Throughout the training program, the participants acquired a greater confidence in this dimension, widening their perception of spirituality.

“I understood that time and space (for spirituality) can be found in daily life” (code 6.10 T1)

“I hadn’t nurtured my spirituality for a long time, and then picked it up again after the course” (code 8.6 T1)

“The course increased my attention to spirituality, also applying it to personal difficulties as I would have done in the past” (code 10.5 T1)

Most trainees expressed a sense of gratitude for being able to practice and work on self-development.

“Thinking how lucky we are to live our daily lives: that is spirituality” (code 4.15 T2)

“I learned gratitude at the end of the conversation with the other professionals” (code 5.13 T2)

“For me, it was a privilege, a blessing to take this course in the workplace” (code 2.4 T2)

Reflecting on personal spirituality led to the need to keep spiritual awareness high.

“The course kept a strong focus on the human and spiritual dimension” (code 9.15 T2)

On the other hand, the increased familiarity with the topic revealed a phenomenon we called “fatigue/sense of fatigue of care providers” and “difficulty of applying to daily work”.

“I wanted to look deeper into some personal aspects of spirituality, but so far I haven’t been able to” (code 7.30 T2)

“It is challenging to put into practice what I learned in the course” (cod 8.10 T2)

“I struggle to cultivate spirituality on my own” (code 5.27 T2)

Both positive and negative aspects showed, however, an “increased ability to self-reflect” on this dimension and confirmed the theme of the adopted FM.

Analysis of the reflective writing (RW) mostly confirmed the data from the interviews and the FM (see Table 5). Moreover, through this analysis, it was possible to develop a reflection on the various components included in each phase of the training (in brackets the HP code).

**Table 5:** Themes, sub-themes and overarching themes in RWs

**Themes T1 and sub-themes T1**	**Themes T2 and sub-themes T2**	**Themes T3 and sub-themes T3**	**Themes T4 and sub-themes T4**
***Developing closeness with colleagues.*** • Getting to know each other better • Working in pairs leads to openness. • The connection can be fast. • Enrichment through diversity	***Becoming aware of your own spirituality*** • Seeing your own evolution • Recognizing common themes • New reflections • Spirituality as acceptance of oneself and others	***Exploring spiritual practices*** • Recognizing and rediscovering your own range of practices • Recognition of possible multiplicity	***Being in control of the situation*** • Greater self-awareness • Importance of storytelling • Thinking about the past to think about the future
***Practicing listening*** • Experiencing the feeling of being listened to intently • Feeling welcomed • Importance of the absence of feedback • Making room for each other • Learning to value listening to yourself	***Reconnecting with/finding your spirituality in order to help yourself***	***Nourishing spirituality*** • Importance of nurturing spirituality • Reconfirming resolutions/commitments. • Opening a "space" with a partner • Desire for future opportunities to share	***Contextualizing spirituality in the context of work*** • Working on yourself to improve care relationships • Being non-judgmental of patients • Professional as a spiritual healing agent • Team sharing of the theme and of a common language • Greater ease in talking with patients and family members • Improving relationships with colleagues through the habit of non-judgment
		***Identifying listening and talking as spiritual practices***	***Recognizing and welcoming spirituality in others*** • From personal to interpersonal spirituality • Accepting others through spirituality
		***Giving value to emptiness*** • Spirituality as emptiness: accepting this	***Being aware of the approach*** • Match between thought/experience. • Experience of listening and being heard. • Not being a learner/peer exchange
**Overarching themes**			
***Role of others in listening to yourself***	***Role of others in influencing spirituality*** ***Role of others in recognizing one’s own spirituality*** ***Role of others in recognizing oneself and broadening the mind***	***Role of others: discussions with other people broadens the mind*** ***Role of others in self-exploration, for the interweaving of experiences*** ***Role of others in enrichment through diversity***	***Role of others: spirituality passes through the relationship***
***Possible transferability to the care relationship***	***Possible transferability to the care relationship***	***Possible transferability to the care relationship***	***Possible transferability to the care relationship***
***Learning to value your time***	***Desire to spend time rethinking the past and rediscovering spiritual practices***	***Importance of setting aside time: gratitude for feeling justified in spending this time in this way***	***Time needed to listen*** ***Importance of creating moments of emptiness***

After the first RW, trainees felt closer to colleagues.

“My ‘partner’ enriched my point of view because she brought reflections that were different from mine; in any case, it was a way to get to know her better and feel closer to her; there was a nice moment of closeness. (psychologist code 1, T1)”

The active listening exercise was carried out in pairs, and this was gradually recognized as an important tool for spiritual care.

“It was a sort of training on my ability to interact with patients/family members/colleagues” (physician code 3, T1).

After the second RW, an awareness of the HPs’ relationship with spirituality and with spiritual references emerged. This was often a new reflection; at other times, participants recovered some previous knowledge they had set aside.

“I believe that the objective of the questions posed by the speakers, including about childhood, was to trigger something in me to rediscover and recognize this part of me.”

The transferability to clinical practice was recognized, for instance, when thinking about using the same questions used in the training with patients.

“I always find the questions proposed to us each time very interesting, for myself and patients” (nurse, code 10 T2).

Both the first and the second RW showed an impact on Moore’s Level 3B, based on the self-assessment of participants. This subject of transferability is an overarching theme.

In the third RW, the need to explore new spiritual practices emerged, which was consistent with theme 3 from the interviews (“Spirituality and the need for nourishment”).

“What I’ll take away is the fact that there are so many activities I can use to increase my spirituality and that I can ‘create’ others to help me to find answers to my questions about the meaning of life”. (nurse, code 5 T3).

Finally, after the fourth RW, self-reflection, and awareness of one’s spirituality was achieved through “closing the circle” and opening the path to continue the HPs’ journey.

“It was an important moment to close the circle and take stock of what it meant to me and at the same time open up new perspectives on how to use these “breakthroughs” of mine in the future” (bioethicist, code 7 T4).

The need to explore a spiritual dimension in daily work was also developed, both through sharing with colleagues and by perceiving more confidence in meetings with patients and families.

“The most practical lesson I will take home is the realization that every day, in every moment, we have to work on our spirituality and that of the people around us, often even without fully realizing how we can be a tool for others to reflect on this aspect too, which I consider very personal and intimate.” (physician, code 4 T4).

“I have certainly become more open to the possibility of spiritual care in my work as a part of the in-hospital palliative care team” (physician, code 11 T4).

In addition to these, the RWs highlighted 3 overarching themes.

- *1-. Value of time dedicated to spirituality* – Several participants felt that one of the most important components of the training program was “simply” the possibility of feeling justified and even obliged to take time out from the daily work tasks to work on their spirituality.
  “Having reflected on the things that emerged, and having understood some parts of myself better, I feel that spirituality was always part of my life. Looking at this in more depth through a dedicated day with my team was enriching and moving.” (psychologist code 1 T1)”
- *2-. The role of others in spirituality*– At T1, the active listening exercise led the participants to reflect on and understand the pivotal role of others (in this case their colleagues) in working on one’s spirituality. In the second and third RWs, many commented on coming to see diversity as an enrichment and the value of the absence of judgment. Lastly, in the final RW, connection with others was often described as a spiritual practice. This led many participants to establish a “space” to develop spirituality with their professional partners.
  “A space has been created, with my partner, which we can enter and stay in times of need. (nurse code 10 T3).”
- *3-. Relevance of spirituality to relationships/transferability to care relationships –* The act of practicing spirituality through the training (active listening, reconnecting to one’s roots and one’s spiritual references, openness towards other practices, and an attitude of non-judgment and welcoming diversity) led participants to see themselves as spiritual care tools, to feel greater ease in interviews, to share this dimension in a team and even to improve team relationships.
-
  “I believe it was useful to me and achieved the objective of making me move from personal to interpersonal spirituality, with colleagues and with patients (physician code 11 T4).

## Discussion

This study focused on the development of a spiritual training program for specialized PC professionals working within a hospital, the different components of the intervention, and its preliminary assessment.

The program was completed as established for all participants and most of the components were highly appreciated. According to these, the results of this study suggest the feasibility of a training course with some corrections, both regarding the components of the training and organizational issues. The RW as a tool for personal reflection could be proposed without making it compulsory, given the different backgrounds of the participants and the different specific sensitivities, and we could avoid online sessions.

Having dedicated time and the support of the hospital management for these types of courses guarantees their implementation even in a hospital setting. Albuquerque et al. [26] already noted that organizational culture is a determining factor in working on spirituality.

Many participants suggested that it was important to have feedback at the end of the day from the spiritual assistants and a monthly discussion meeting with them; the introduction of a monthly discussion with spiritual assistants for the maintenance of acquired skills is a component to be introduced.

As the qualitative data shows, the spiritual dimension of a specialist multidisciplinary PCU in a hospital was, first, reawakened by the course; that is, spirituality was present and already being cultivated by trainees, but the course helped everyone to recognize their spiritual roots and they consciously expanded the connection to patients and colleagues. The course enriched the participants with a broader search for meaning, transcendence, and a more pervasive vision of the spiritual dimension of the group. The themes of gratitude, greater attention to the spiritual dimension, and a greater need for sharing with colleagues emerged after the course. Being a course that worked on everyone’s spirituality did not have a direct impact on patient care, but it certainly changed the way we deal with each other, being careful to talk about this dimension in team meetings.

Our study followed the recognized recommendations on spiritual care teams (SCTs) by recent studies; the main objectives of spiritual care training should be developing trainees’ sensitivity towards their spirituality, clarifying the role of spirituality in healthcare, and preparing trainees for spiritual interactions [7, 27–31].

If we consider the five key areas for developing skills in intrapersonal spirituality identified by Jones et al. in their systematic review [7], we can show how Moore et al.’s assessment, our results, and the spirituality skills intertwined in this training program.

The five key areas are the levels of competence identified for intrapersonal spirituality.

i) understanding the concept of spirituality.
ii) recognizing the importance of the spiritual dimension in patient care.
iii) gaining awareness of the importance of understanding one’s spirituality before addressing the spiritual needs of others.
iv) valuing the importance of self-care.
v) an increasing ability to self-reflect.

Points i) and ii) relate to LEVEL 3A (the degree to which participants state what the training intended them to learn) of Moore et al., while points iii), iv), and v) concern LEVEL 3B (the degree to which participants state how to do what the training intended them to learn how to do).

We identified a theme that we called *self-reflection on one’s spirituality* corresponding to an “increased ability to self-reflect”.

This level was achieved after passing through *What is Spirituality?* (corresponding to 2 FM themes “Understanding the concept of spirituality” and “Gaining awareness of the importance of understanding one’s spirituality before addressing the spiritual needs of others”), *Getting spiritual experience at work (*corresponding to “Recognizing the importance of the spiritual dimension in patient care”) and *Spirituality and the need for nourishment* (corresponding to **“**Valuing the importance of self-care”).

The data showed that there was an impact on Moore’s Level 3B. An increased ability to self-reflect on spirituality and to value the importance of self-care was identified.

Regaining contact with one’s spirituality and broadening the perception of spirituality itself has led to a greater need for nourishment, a greater need for sharing with colleagues, and a need for spiritual care tools. The trainees went from intrapersonal spirituality to interpersonal spirituality (engagement with the other person’s spirituality, acknowledging their unique spiritual and cultural worldviews, beliefs, and practices), with colleagues, patients, and people close to them. This result was confirmed in the Daudt et al. study, where the theme of connection and team building was witnessed by EDUC participants working in a hospice [31].

Moreover, our results regarding the experiential learning of real-life spiritual interactions between multidisciplinary peers with SCPs trained in deep listening, as well as silence and establishing a connection as a spiritual care tool in a work environment were confirmed in other studies by [31]. In addition, it was also recognized how the training was not, in fact, a traditional, notional form of training but rather a practice of spiritual guidance. This aspect is important because it is what is also required of HPs in relation to patients and families.

### Strengths and limitations

We think that the greatest strength of our study is the data triangulation (i.e., combining interviews, reflective journals, and feedback), which increased the validity of the study findings [32].

Moreover, data collection (feedback on the training components) took place at the same time as the training, allowing the joint creation of the training course between teachers and trainees. The FM allowed us to connect and triangulate data within the analysis.

Another major strength of our training program was the multidisciplinary aspect; that is, that spiritual care can be delivered, to some extent, by all healthcare professionals. For this reason, interdisciplinary education in spiritual care delivery has been promoted through the interprofessional spiritual care model [6, 28]; the course contributes to the development of a common language between different disciplines on spirituality, as also suggested by the significant convergence between *connection* and *gratitude*, the most frequent words present in the RW and semi-structured interviews at T2.

On the other hand, the small and homogeneous nature of the group represents a limitation to the generalizations of the findings, and the significant interest of the participants in spiritual issues could be biased. A performance assessment of participants’ behaviors has not yet been carried out (Moore’s Level IV-V) as recommended by the literature, but the study aimed to work on the personal spiritual dimension, which is independent of work performance.

## Conclusions

The training course was performed as established and was greatly appreciated. It was feasible and acceptable for care providers working in an Italian hospital setting.

Reflecting on their spirituality spontaneously raised the need for HPs to have spiritual tools to care for patients. Having time dedicated to spirituality and the ongoing mentorship of SCPs were suggested as key elements for success and support by management. The difficulty of finding space and time to develop this dimension during work arose, and the need to provide opportunities consciously and systematically for this kind of work in a healthcare environment was suggested. Not having professional figures such as SCPs in the facility is a major limitation to the reproducibility of the course. We are trying to raise awareness and gather in a research group of people competent on the subject from our context (such as nurses, chaplains, nuns, psychologists, counselors, etc.).

### Supplementary Information

**Additional file 1.**

**Additional file 2.**

**Additional file 3.**

**Additional file 4.**

## Data Availability

The data presented in this study are available on request from the corresponding author. The data are not publicly available for ethical reasons.

## Abbreviations

HP: Health Professional
SCP: Spiritual Care Professional
STP: Spiritual Training Program
RW: Reflective Writing
EAPC: European Association of Palliative Care
ISPEC: Interprofessional Spiritual Care Education Curriculum
OECI: Organization of European Cancer Institutes
PCU: Palliative Care Unit
BU: Bioethics Unit
ICU: Intensive Care Unit
MRC: Medical Research Council
FM: Framework method
SPCS: Specialist Palliative Care Service
SC: Spiritual Care
SCT: Spiritual Care Training
PC: Palliative Care

## References

[1] Radbruch L, De Lima L, Knaul F, Wenk R, Ali Z, Bhatnaghar S (2020). Redefining palliative care-a new consensus-based definition. J Pain Symptom Manage.

[2] Bickel KE, McNiff K, Buss MK, Kamal A, Lupu D, Abernethy AP (2016). Defining high-quality palliative care in oncology practice: an American society of clinical oncology/American academy of hospice and palliative medicine guidance statement. J Oncol Pract.

[3] Cobb MR, Puchalski CM, Rumbold B. Oxford textbook of spirituality in healthcare. Oxford University Press; 2012. 10.1093/med/9780199571390.001.0001. [cited 2023 Nov 23].

[4] Best M, Leget C, Goodhead A, Paal P (2020). An EAPC white paper on multi-disciplinary education for spiritual care in palliative care. BMC Palliat Care.

[5] McGee J, Palmer Kelly E, Kelly-Brown J, Stevens E, Waterman BL, Pawlik TM. Assessing the impact of provider training and perceived barriers on the provision of spiritual care: a mixed methods study. J Cancer Educ. 2021. 10.1007/s13187-021-02115-x. 10.1007/s13187-021-02115-x 34767182

[6] Puchalski C, Jafari N, Buller H, Haythorn T, Jacobs C, Ferrell B (2020). Interprofessional spiritual care education curriculum: a milestone toward the provision of spiritual care. J Palliat Med.

[7] Jones KF, Paal P, Symons X, Best MC (2021). The content, teaching methods and effectiveness of spiritual care training for healthcare professionals: a mixed-methods systematic review. J Pain Symptom Manage.

[8] Baile WF, De Panfilis L, Tanzi S, Moroni M, Walters R, Biasco G (2012). Using sociodrama and psychodrama to teach communication in end-of-life care. J Palliat Med.

[9] Landreville J, Cheung W, Frank J, Richardson D (2019). A definition for coaching in medical education. Can Med Educ J.

[10] Farrell L, Cuncic C, Hartford W, Hatala R, Ajjawi R (2023). Goal co-construction and dialogue in an internal medicine longitudinal coaching programme. Med Educ.

[11] McHugh SK, Lawton R, O’Hara JK, Sheard L (2020). Does team reflexivity impact teamwork and communication in interprofessional hospital-based healthcare teams? A systematic review and narrative synthesis. BMJ Qual Saf.

[12] Lovell B (2018). What do we know about coaching in medical education? A literature review. Med Educ.

[13] Rees CE, Nguyen VNB, Ottrey E, Davis C, Pope K, Lee S (2022). The effectiveness of extended-duration supervision training for nurses and allied health professionals: a realist evaluation. Nurse Educ Today.

[14] Jones KF, Pryor J, Care-Unger C, Simpson G (2022). “Spirituality is everybody’s business”: an exploration of the impact of spiritual care training upon the perceptions and practice of rehabilitation professionals. Disabil Rehabil.

[15] Attard DJ, Ross DL, Weeks KW (2019). Developing a spiritual care competency framework for pre-registration nurses and midwives. Nurse Educ Pract.

[16] Baldacchino DR (2011). Teaching on spiritual care: the perceived impact on qualified nurses. Nurse Educ Pract.

[17] Murray RP, Dunn KS (2017). Assessing nurses’ knowledge of spiritual care practices before and after an educational workshop. J Contin Educ Nurs.

[18] Vlasblom JP, van der Steen JT, Knol DL, Jochemsen H (2011). Effects of a spiritual care training for nurses. Nurse Educ Today.

[19] van de Geer J, Veeger N, Groot M, Zock H, Leget C, Prins J (2018). Multidisciplinary training on spiritual care for patients in palliative care trajectories improves the attitudes and competencies of hospital medical staff: results of a quasi-experimental study. Am J Hosp Palliat Care.

[20] Moore DEJ, Green JS, Gallis HA (2009). Achieving desired results and improved outcomes: integrating planning and assessment throughout learning activities. J Contin Educ Health Prof.

[21] Campbell M, Fitzpatrick R, Haines A, Kinmonth AL, Sandercock P, Spiegelhalter D (2000). Framework for design and evaluation of complex interventions to improve health. BMJ.

[22] Miccinesi G, Claudio Ritossa, Luca Manfredini, Giovanni Zaninetta, Cagna M, Filippo Laurenti, et al. Core Curriculum dell’Assistente Spirituale in Cure Palliative. 2022.

[23] Puchalski CM (2014). The FICA spiritual history tool #274. J Palliat Med.

[24] Luigina Mortari. Apprendere dall’esperienza. Il pensare riflessivo nella formazione. Roma: Carrocci; 2003.

[25] Zhang W, Creswell J (2013). The use of ‘mixing’ procedure of mixed methods in health services research. Med Care.

[26] Faro Albuquerque I, Campos Cunha R, Dias Martins L, Brito SA (2014). Primary health care services: workplace spirituality and organizational performance. J Organ Chang Manag.

[27] Paal P, Helo Y, Frick E (2015). Spiritual care training provided to healthcare professionals: a systematic review. J Pastoral Care Counsel.

[28] Balboni MJ, Puchalski CM, Peteet JR (2014). The relationship between medicine, spirituality and religion: three models for integration. J Relig Health.

[29] Chahrour WH, Hvidt NC, Hvidt EA, Viftrup DT (2021). Learning to care for the spirit of dying patients: the impact of spiritual care training in a hospice-setting. BMC Palliat Care.

[30] Hvidt EA, Ammentorp J, Søndergaard J, Timmermann C, Hansen DG, Hvidt NC (2018). Developing and evaluating a course programme to enhance existential communication with cancer patients in general practice. Scand J Prim Health Care.

[31] Daudt H, d’Archangelo M, Duquette D (2019). Spiritual care training in healthcare: does it really have an impact?. Palliat Support Care.

[32] Carter N, Bryant-Lukosius D, DiCenso A, Blythe J, Neville AJ (2014). The use of triangulation in qualitative research. Oncol Nurs Forum.

