# Supplementary material for: Experiential training course on spirituality for multidisciplinary palliative care teams in a hospital setting: a feasibility study
Source: BMC Palliat Care. 2024 Feb 10;23:38. doi: 10.1186/s12904-024-01341-6 (PMC10858494; doi:10.1186/s12904-024-01341-6)
Supplement: Supplementary file 1 — Additional file 1. [file 12904_2024_1341_MOESM1_ESM.doc]

**Training course on Spirituality for Health Professionals**

***Semi-structured interview at T0***

***Instructions for the interviewer***

***Before starting the interview, request consent and have the appropriate form signed.***

***The interview is focused on carrying out a self-assessment of one's spiritual dimension and consists of four sections aimed at exploring four fundamental themes:***

*1. What is spirituality?*

*2. How to recognize one's own spirituality*

*3. How to nurture and develop your own spirituality*

*4. Expectations for the training program*

**Some sample questions are given for each area.**

In conducting the interview, the interviewer should refer to the questions indicated. Nonetheless, in order for the interview to go smoothly, it is essential that it takes place in a climate based on non-judgmental listening and mutual trust. For this reason, the questions can also be asked in a different order from that shown in the text and, in any case, they should be used as much as possible starting from what was expressed verbally and non-verbally by the interviewee. For the same reason, the questions can be varied and reformulated as needed on the basis of what happens during the conversation.

At the end of the interview, check that you have covered all the topics specified.

**Introduction to the interview**

Before starting the interview proper, it is helpful to try to put the interviewee at ease as much as possible, thanking him/her for having accepted the invitation and being willing to provide clarifications.

Examples of questions:

Thank you for being here.

With respect to the email you received, is there anything that is not clear?

**Opening question**

**Thinking about the spiritual dimension of being a healthcare professional, what would you say are the elements of this dimension in your experience?**

**What is spirituality for you?**

These questions are a guide to starting a conversation about the person's spirituality in the most respectful way possible.

Questions can be modified to suit each respondent's personal situation.

Sample questions

And thinking about your personal spirituality, would you like to try to describe what it is?

(Could you give me an example of what you described?)

Can you tell me how you feel about talking about your spirituality?

**How to recognize one's own spirituality**

These questions help to understand how the interviewees can be more or less able to make contact with their own spirituality and to recognize its constituents.

The answers to these questions can help to identify the level of contact with the respondent’s innermost dimensions and possibly the level of comfort he/she experiences talking about spirituality or living it consciously**.**

Examples of questions:

In this period of your life, in which moments do you try to get closer to your spirituality?

How do you approach your spirituality?

How do you experience these moments of attention to your spiritual dimension?

**How to nurture and develop your own spirituality**

These questions help to understand how and if the person is willing to take care of and develop their own spiritual dimension and what methods they may prefer.

**Examples of questions**

How can you contribute to developing your spirituality?

If it's something you're already doing, could you tell me how it’s working?

What results, if any, do you feel you have achieved?

**Training expectations**

This question allows us to understand how the participants approach this training proposal, which offers them an individual path of reflection.

*Question example:*

*Could you tell me how you felt about the proposal for a training course on spirituality for health professionals? What are your expectations?*

**Final question:**

Example:

Is there anything else that came to mind during our interview?

**Closing the interview**

To conclude the interview, thank the interviewee and check his/her availability for a subsequent meeting.
