# Supplementary material for: Experiential training course on spirituality for multidisciplinary palliative care teams in a hospital setting: a feasibility study
Source: BMC Palliat Care. 2024 Feb 10;23:38. doi: 10.1186/s12904-024-01341-6 (PMC10858494; doi:10.1186/s12904-024-01341-6)
Supplement: Supplementary file 2 — Additional file 2. [file 12904_2024_1341_MOESM2_ESM.doc]

**Training course on Spirituality for Health Professionals**

**Semi-structured interview at T1**

***Instructions for the interviewer***

*The T1 interview is carried out after the training intervention comprising interviews, writings and reflections.*

*The interview is focused on carrying out a self-assessment of one's spiritual dimension and consists of five sections aimed at exploring five fundamental themes:*

*1. What is spirituality?*

*2. How to recognize one's own spirituality*

*3. How to nurture and develop your own spirituality*

*4. Expectations for the training course*

*5. Evaluation of the individual components of the training course*

**Opening question for the interview**

After the experience you have had, thinking about the spiritual dimension of being a healthcare professional, what would you say are the elements of this dimension?

**What is spirituality for you?**

After participating in this training, thinking about your own experience, could you describe what spirituality is for you?

(Could you give me an example of what you described?)

Can you tell me how you feel about talking about your spirituality?

**How to recognize your own spirituality**

After participating in this training, in which moments do you try to get closer to your spirituality?

How do you approach your spirituality?

How do you experience these moments of attention to your spiritual dimension?

**How to nurture and develop your own spirituality**

After the training experience, how do you think you can contribute to developing your spirituality?

If it's something you're already doing, could you tell me how you're doing?

What results, if any, do you feel you have achieved?

**Training expectations**

Would you like to tell me how you experienced the training course on spirituality for professionals? What do you think its consequences might be?

**Evaluation of the individual components of the training course**

Could you tell me what was (or what were) the most significant moment(s) of the course for you? Could you explain why?

Could you give an example?

**Final question:**

Example:

Is there anything else that came to mind during our interview?

**Closing the interview**

To conclude the interview, thank the interviewee and check his/her for a subsequent meeting.
