# Supplementary material for: Experiential training course on spirituality for multidisciplinary palliative care teams in a hospital setting: a feasibility study
Source: BMC Palliat Care. 2024 Feb 10;23:38. doi: 10.1186/s12904-024-01341-6 (PMC10858494; doi:10.1186/s12904-024-01341-6)
Supplement: Supplementary file 3 — Additional file 3. [file 12904_2024_1341_MOESM3_ESM.doc]

**Training course on Spirituality for Health Professionals**

***Semi-structured interview at T2***

Instructions for the interviewer

The T2 interview is carried out at the end of the training course, including the follow-up.

The interview is focused on carrying out a self-assessment of one's spiritual dimension and consists of four sections aimed at exploring four fundamental themes:

1. What is spirituality?

2. How to recognize one's own spirituality

3. How to nurture and develop your own spirituality

4. Effects of the training course

**Opening question for the interview**

Thinking about your training experience, how would you express it with keywords or an image or a metaphor?

**What is spirituality for you?**

Thinking about the training carried out so far, would you like to try to describe what spirituality is for you?

(Could you give me an example of what you described?)

Can you tell me how you feel now about talking about your spirituality?

**How to recognize your own spirituality**

After the training experience, in which moments do you try to get closer to your spirituality?

How do you approach your spirituality?

How do you experience these moments of attention to your spiritual dimension?

**How to nurture and develop your own spirituality**

Still thinking about the training experience you had, how do you think you can contribute to developing your spirituality?

If it's something you're already doing, could you tell me how you're doing now?

What results, if any, do you feel you have achieved?

**Effects of the training course**

This question allows us to understand how the participants feel about the experience gained and above all what they consciously took away from it.

**Question example:**

Could you tell me how you lived the experience of this training course on spirituality for professionals? What skills do you think you have acquired that you didn't have before? Could you give an example?

**Final question:**

Is there anything else that came to mind during our interview?

**Closing the interview**

To conclude the interview, thank the interviewee for their participation.
