# Supplementary material for: Experiential training course on spirituality for multidisciplinary palliative care teams in a hospital setting: a feasibility study
Source: BMC Palliat Care. 2024 Feb 10;23:38. doi: 10.1186/s12904-024-01341-6 (PMC10858494; doi:10.1186/s12904-024-01341-6)
Supplement: Supplementary file 4 — Additional file 4. [file 12904_2024_1341_MOESM4_ESM.doc]

| **CODE** | **AGE** | **GENDER** | **PROFESSION** | **EXPERIENCE IN PC (YEARS)** |
| --- | --- | --- | --- | --- |
| 01 | 40 | F | psychologist | 15 |
| 02 | 45 | F | nurse | 6 |
| 03 | 55 | F | physician | 1.5 |
| 04 | 41 | F | physician | 7 |
| 05 | 43 | F | nurse | 1 |
| 06 | 46 | F | psychologist | 7-8 |
| 07 | 27 | F | bioethicist | 3-4 |
| 08 | 38 | F | bioethicist | 7 |
| 09 | 41 | F | psychologist | 7 |
| 10 | 35 | F | nurse | 5 |
| 11 | 40 | F | physician | 6 |
| 12 | 39 | F | psychologist | 5-6 |

**Appendix 4: participants’ characteristics and their codes**
